# Heart rate variability can clarify students’ level of stress during nursing simulation

**DOI:** 10.1371/journal.pone.0195280

**Published:** 2018-04-05

**Authors:** Natsuki Nakayama, Naoko Arakawa, Harumi Ejiri, Reiko Matsuda, Tsuneko Makino

**Affiliations:** 1 Department of Nursing, Nagoya University Graduate School of Medicine, Nagoya, Aichi, Japan; 2 Department of Nursing, College of Life and Health Sciences, Chubu University, Kasugai, Aichi, Japan; Tokai University, JAPAN

## Abstract

Simulation is regarded as an effective educational method for the delivery of clinical scenarios. However, exposure to unfamiliar environments during simulation can cause excessive stress among students, possibly leading to unnatural speech/behavior and poor skill learning (Yerkes-Dodson’s law). Thus, assessing students’ stress in a simulation can provide educators with a better understanding of their mental state. This study sought to clarify stress changes throughout the progression of the simulation by measuring heart rate variability and students’ subjective reactions in 74 nursing students. Heart rate variability was calculated in terms of its high-frequency (HF) and low-frequency/high-frequency (LF/HF) components during 4 phases—the break, patient care, reporting, and debriefing. Students were interviewed about stress experienced during the simulation. The results showed that HF decreased significantly from the break to the patient care and reporting phases. Furthermore, LF/HF increased significantly from the break to the reporting phases. Approximately 55 students felt stressed during the simulation, 24 of whom felt most stressed during the reporting phase. Therefore, the reporting phase involved high objective and subjective stress. It may be possible that the educator’s evaluative attitude increased students’ stress. Therefore, a stress intervention during the reporting phase might further improve students’ performance during that phase. The debriefing phase did not significantly differ from the break phase for objective stress, and students did not report feeling stressed. Thus, in this phase, they were released from the stress of the reporting phase and the unfamiliar environment. During this phase, they might be able to learn what they could not understand owing to high stress in the patient care and reporting phases. This study provides objective and subjective evidence of students’ stress during simulation, and indicates the necessity of providing support during the reporting phase and the importance of debriefing when using clinical scenarios for teaching clinical skills.

## Introduction

Among critical care providers (physicians, nurse practitioners, physician assistants, and nurses), experience in dealing with patients and situations has been shown to improve patient outcomes [1]. In particular, for junior nursing students, clinical experiences are an important element of the nursing profession [2]. Nursing students tend to believe that the clinical environment is the most influential educational component for acquiring nursing knowledge and skills [3]. However, a problem that students may face in their training, which has the potential to negatively affect their training outcomes, is the mental disturbance caused by exposure to unfamiliar environments or stress (e.g., clinical training at hospitals) [4].

Simulation is a learning method that involves the use of high-fidelity manikins that enable nurses to experience diverse clinical settings without threatening patients’ safety. Simulation has been utilized in the training of nursing/pharmacology students, as well as in the continued education of nurses, surgeons, physicians, and others working at the forefront of critical care [5–7]. It is highly appreciated and has been broadly accepted by nursing educators. Shreeve (2008) has demonstrated that learning methods based on actual experience, such as simulation, can equip students with new capabilities (e.g., clinical thinking and clinical judgment) that cannot be effectively taught with, but can complement, lecture-based learning [8].

McEwen and Gianaros (2010) proposed that human performance in response to stress falls on a curve, whereby low or high stress affects performance [9]. The simulation environments that students are unfamiliar with, particularly those suffused with various high-fidelity manikins and electronic devices (computer monitors), can cause stress among students, possibly leading to unnatural speech/behavior and poor skill learning [10]. Students’ heart rate has been shown to rise, which was interpreted as the result of mental stress caused by environmental changes during simulation [11]. The simulation itself can also serve as a source of stress, sometimes even leading to the appearance of symptoms of posttraumatic stress disorder, which is called “simulator sickness,” as was observed during stress in a simulation study by Biernacki et al. regarding nursing students [12]. Individuals vary substantially in their threshold for stress and ability to cope with environmental changes. Educators need to understand students’ stress level and the stress changes accompanying simulation, as this enables them to provide more effective educational support. Several objective methods of evaluating students’ stress have been employed in various research fields.

One of the tools available for objective evaluations of stress is heart rate variability (HRV). This is calculated from the changes in R-wave intervals in consecutive normal ECG signals measured by the Holter electrocardiography system. HRV is widely accepted as an indicator of autonomic nervous activity [13]. It has already been clarified in previous research that it is possible to measure stress using HRV [14,15]. According to frequency analysis of HRV (MemCalc GMS), it can be divided into two components: low-frequency (LF; 0.04–0.15 Hz) and high-frequency (HF; 0.15–0.4 Hz). The HF component is an indicator of parasympathetic nervous system activity, whereas the LF component divided by the HF component (LF/HF) serves as an indicator of sympathetic nervous system activity [16]. Sympathetic nervous system activity is related to increased stress levels, whereas parasympathetic nervous activity is related to relief from stress. Thus, assessing this physiological response in students might provide educators with a better understanding of their stress state. Providing an objective measure of their stress might also help students better understand their own unconscious selves and control their emotions. The purpose of this study was to clarify the stress changes throughout the simulation by using HRV and the subjective reactions of students.

## Methods

### Description of the postoperative nursing simulation

The purpose of this simulation was to allow students to observe postoperative patients and learn to assess patients accurately using available information on them. The educator informed students that they would see a patient one hour after the patient’s tracheal tube had been removed within the operating room. Students were further instructed to observe the patient’s condition and position of the patient’s body, and to pay attention to the patient’s respiratory condition. In addition, the students were asked to conduct a medical interview with the manikin. After explaining this process to the students, the educator was waiting in another room next door. The educator observed the students through a one-way mirror window in the next room from which the educator could see the students, but the students could not see the educator. The educator observed students’ performance on the observation of respiratory condition, which included confirmation of position of the patient’s body, oxygen dose, auscultation of respiratory sound, thorax movement, respiratory frequency, respiratory pattern, and respiratory rhythm. The simulation was progressed into the following phases: 1). introduction (5 minutes; students were given basic information on the patient and setting); 2). a 5-minute break (hereafter, “the break”); 3). patient care (about 10 minutes; the student examined the manikin through auscultation, inspection, and palpation); 4). reporting (5 minutes; student reported on patient care); and 5). debriefing (10 minutes). In the patient care phase, they mainly confirmed the patient’s breathing frequency, breathing sound, cyanosis, and peripheral circulation. In addition, they confirmed that the oxygen tube was not bent and that the dose of oxygen was correct. The debriefing was conducted in a separate room from the patient’s room and was based on the following five guidelines by Decker et al.: (1) facilitated by an individual competent in the debriefing process; (2) conducted in an environment conducive to learning and that supports confidentiality, trust, open communication, self-analysis, and reflection; (3) facilitated by a person(s) who observed the simulated experience; (4) based on a structured framework for debriefing; and (5) congruent with the students’ objectives and outcomes of the simulation-based learning experience [17]. The educator gave feedback about the patient care phase for students. The simulation lasted for about 30 minutes from introduction to debriefing (Table 1).

**Table 1:** Simulation phases.

Phase	Minutes	Contents
1) Introduction	5	Students were given basic information on the patient and setting
2) Break	5	Students were given a 5-minute break
3) Patient care	10	The student examined the manikin through auscultation, inspection, and palpation
4) Reporting	5	Student reported on patient care
5) Debriefing	10	Debriefing

### Simulation scenario

A high-fidelity manikin was installed within the patient’s room. The temperature, relative humidity, and illumination conditions were controlled. The patient was simulated using a life-size manikin for advanced life support training (Laerdal Co., Ltd). This manikin is capable of issuing (and attenuating) a breathing sound and can be set to have specific a heart rate, blood pressure, and respiratory rate in realistic scenarios. The patient simulated was a 53-year-old male who had undergone total gastrectomy for the treatment of gastric cancer. The scenario was set such that the patient’s tracheal tube had been removed within the operating room one hour before the student saw him. The patient had fully recovered consciousness after the operation and was outfitted with a central venous catheter, an oxygen mask with a flow of 3 liters of per minute, a nasogastric tube, a urethral catheter, a sheet of gauze for protection of the celiotomy wound, an indwelling abdominal drain, and an electrocardiogram monitor. We set the manikin’s physiological parameters as follows (heart rate, 62–70 bpm; blood pressure, 120–128/66–70 mmHg; and respiratory rate, 16–20 min).

### Students

Third-year students enrolled in a 4-year nursing university course were recruited as volunteers in this study. All students had acquired the credits for all lecture-based lessons before participation in the study. None of the students had used a high-fidelity manikin before. They had two weeks’ experience of practicing in a hospital setting before this study, during which they had only taken charge of a single patient. None of them had begun surgical nursing practice, and all were unfamiliar with the observation of a real surgical patient. Therefore, a simple and easy-to-resolve scenario was required. Students with hypertension, heart disease, diabetes mellitus, or kidney disease, or who were receiving routine oral medication, were excluded from this study. All students were advised to avoid consuming alcohol and caffeine the day before the study and to sleep well on the night before. Overall, 74 eligible students who provided informed consent for participation and were given an explanation of the ethical considerations enrolled in the study.

### Data collection

Before students entered the patient’s room, the researcher connected students to a Holter electrocardiography system (GLLERT Lab Tech Co., Ltd.) and then gave students an explanation of the setting of the simulation. They were also given a 5-minute break (the break phase) to stabilize their autonomic nervous system. During the break phase, students were advised to take deep breaths while sitting on the chair in a quiet room with the curtains drawn. Five minutes later, students entered the patients’ room with the educator to begin the patient care phase. This phase ended when the student said “completed.” Upon completion of patient care, students reported to the educator what they had observed and assessed with regard to the patient’s condition (the reporting phase). The educator observed the students in a booth connected to the patients’ room, which was blinded from view while in the patients’ room. After being disconnected from the Holter system, the student was interviewed about his/her reflections on the simulation, with emphasis being put on what the student had said of the simulation.

### Analytical methods

The Holter system records were used to calculate heart rate, as well as the HF and LF/HF components of HRV. The simulation was divided into five phases, although HRV was only compared between the following four: (1) break, (2) patient care, (3) reporting, and (4) debriefing. Significant differences in heart rate and the HRV components were determined using the Mann-Whitney U-test with SPSS Statistics 22 (IBM Corp., Armonk, NY). The statistical significance level was set at *p* < 0.05. In addition, we analyzed the meaning of students’ remarks and counted the number of occurrences of certain remarks.

### Ethical considerations

Each student received an explanation from the researcher about the ethical considerations related to the study, including the freedom of the student to participate in the study at her/his own discretion and to cancel participation at any time after the start of the study, and the fact that participation (or lack thereof) would have no influence on the student’s school performance. If students considered these terms acceptable, they were asked to give their written consent. This study was carried out with the approval of the Research Ethics Committee at Chubu University to which the researcher belonged.

## Results

**Fig 1:**
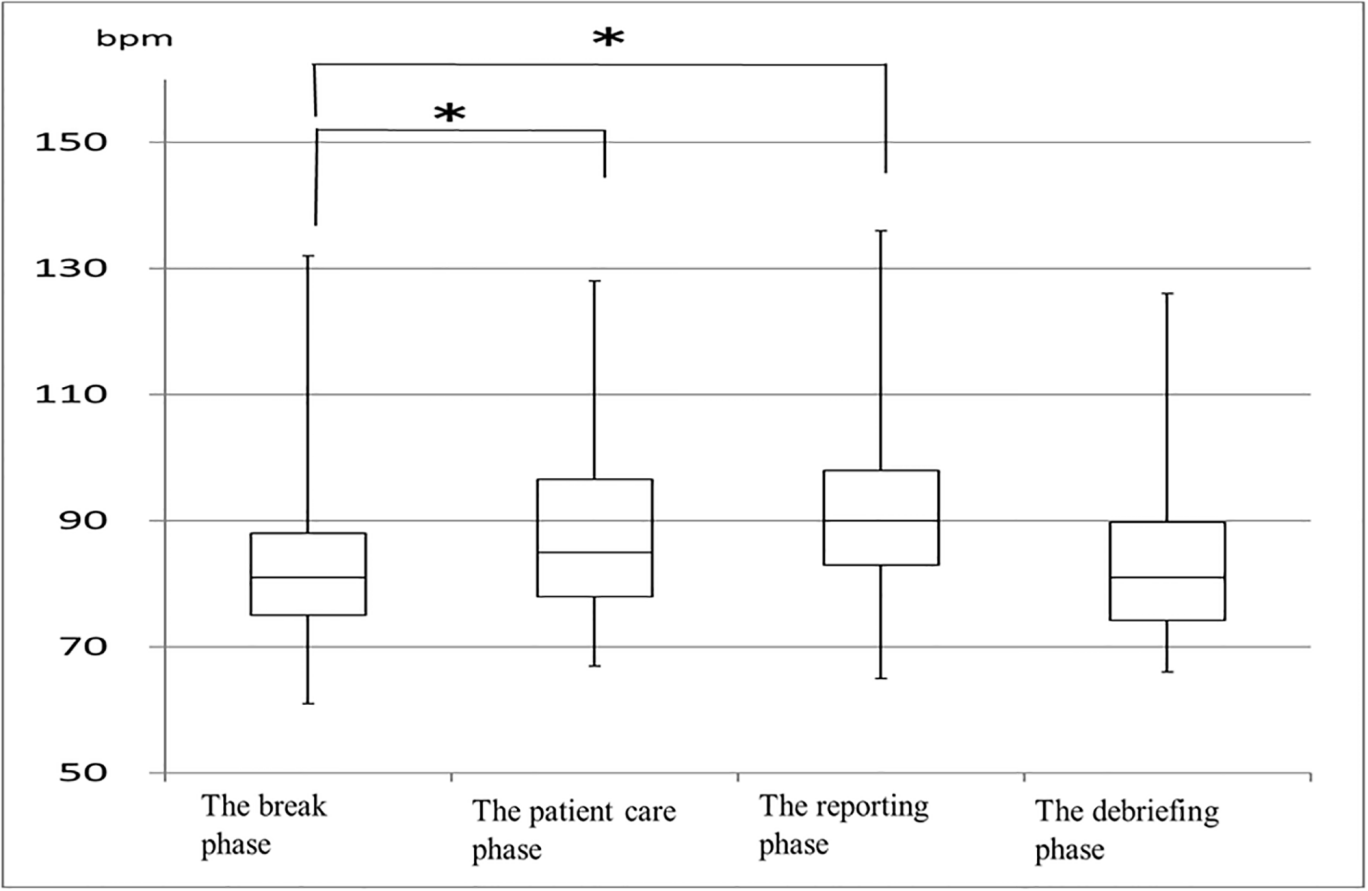
Changes in heart rate at each phase (n = 74) *p < 0.05.

**Fig 2:**
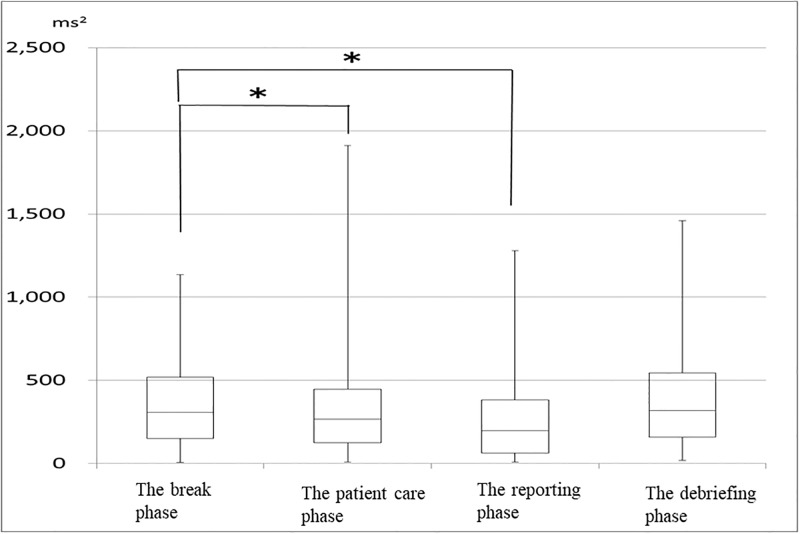
Changes in high frequency component at each phase (n = 74) *p < 0.05.

**Fig 3:**
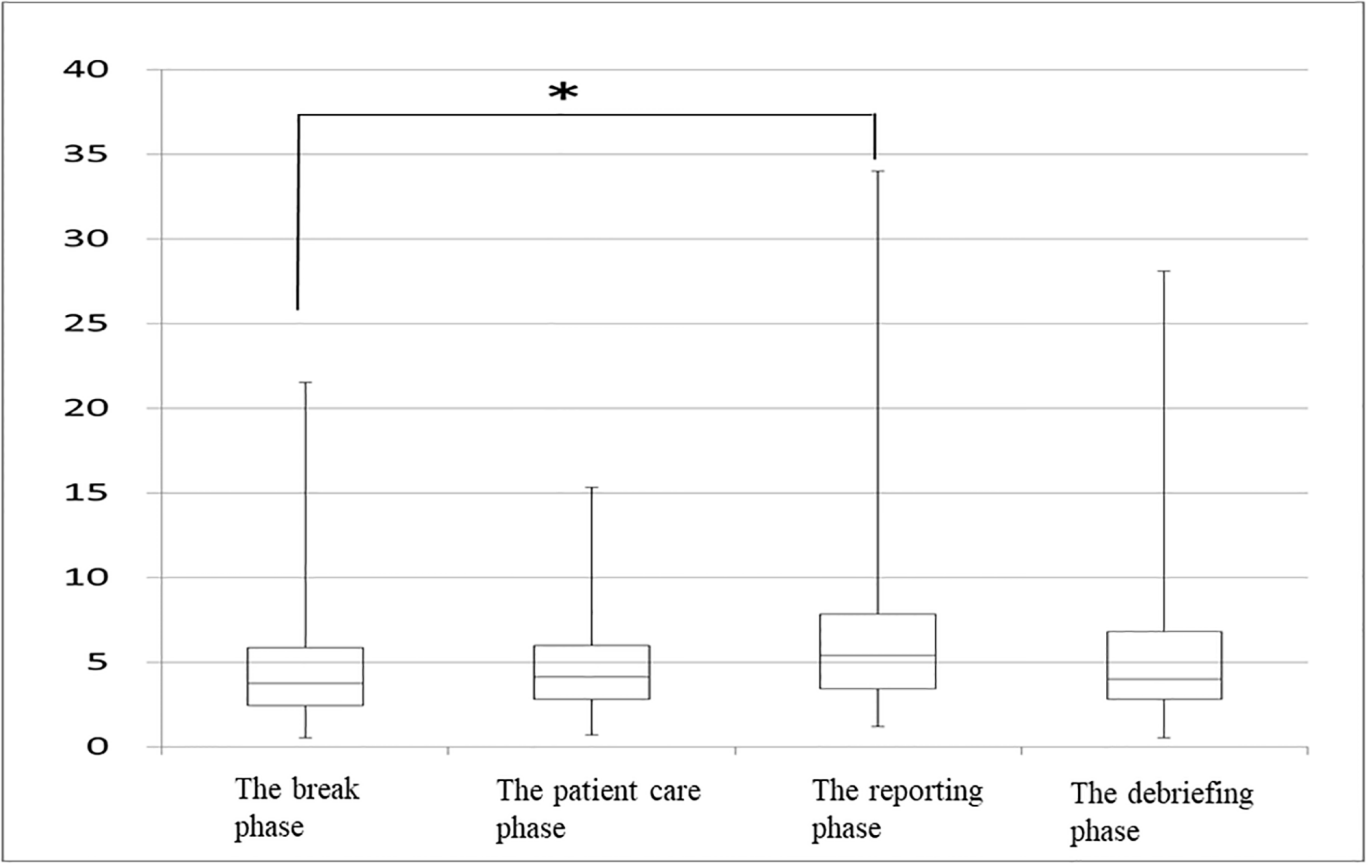
Changes in low frequency/high frequency component at each phase (n = 74) *p < 0.05.

The mean age of students was 21 (20–27) years. Only 4% (n = 3) of the 74 students were male. Heart rate increased significantly from the break phase to the patient care phase (81 [61–132] bpm, 85 [67–128] bpm; *p* < 0.0001). In addition, heart rate increased significantly from the break phase to the reporting phase (81 [61–132] bpm, 90 [65–136] bpm; *p* < 0.0001; Fig 1). The HF component of HRV decreased significantly from the break phase to the patient care phase (308 [5–1136] mm^2^, 266 [9–1911] mm^2^; *p* = 0.001). In addition, HF decreased significantly from the break phase to the reporting phase (308 [5–1136] mm^2^, 196 [8–1279] mm^2^; *p* = 0.0001; Fig 2). LF/HF increased from the break phase (3.7 [0.5–21.5]) to the patient care phase (4.1 [0.7–15.3]; *p* = 0.623). LF/HF increased significantly from the break phase to the reporting phase (3.7 [0.5–21.5], 5.4 [1.2–34.0]; *p* = .0001; Fig 3). LF/HF increased marginally from the break phase to the debriefing phase (3.7 [0.5–21.5], 4.8 [1.6–13.7]; *p* = 0.064), though this result was not significant.

As for the interview results (Table 2), 55 students out of the total 74 said that they felt stress during the simulation. They reported the following:

“*When I entered patient’s room, I could not understand what I should do*.”

“*I was anxious that I had to judge the patient’ state by myself*.”

“*I felt stressed; I felt like I was being watched by the educator*.”

**Table 2:** Students’ reports on stress during the simulation (Multiple answers possible).

**I felt stressed**.	n
I was worried whether I was able to do it properly.	30
I was anxious that I was being watched by the educator.	16
I was not confident.	10
I did not know what to do first.	6
Because the simulator was real.	5
Because there was nobody.	4
**I did not feel stress**.	
Because it was a doll.	11
Because there was no one.	3
Because the simulator was not real.	3
Because it was not a hospital.	1
**I couldn’t recognize whether I felt stressed**.	1

**Fig 4:**
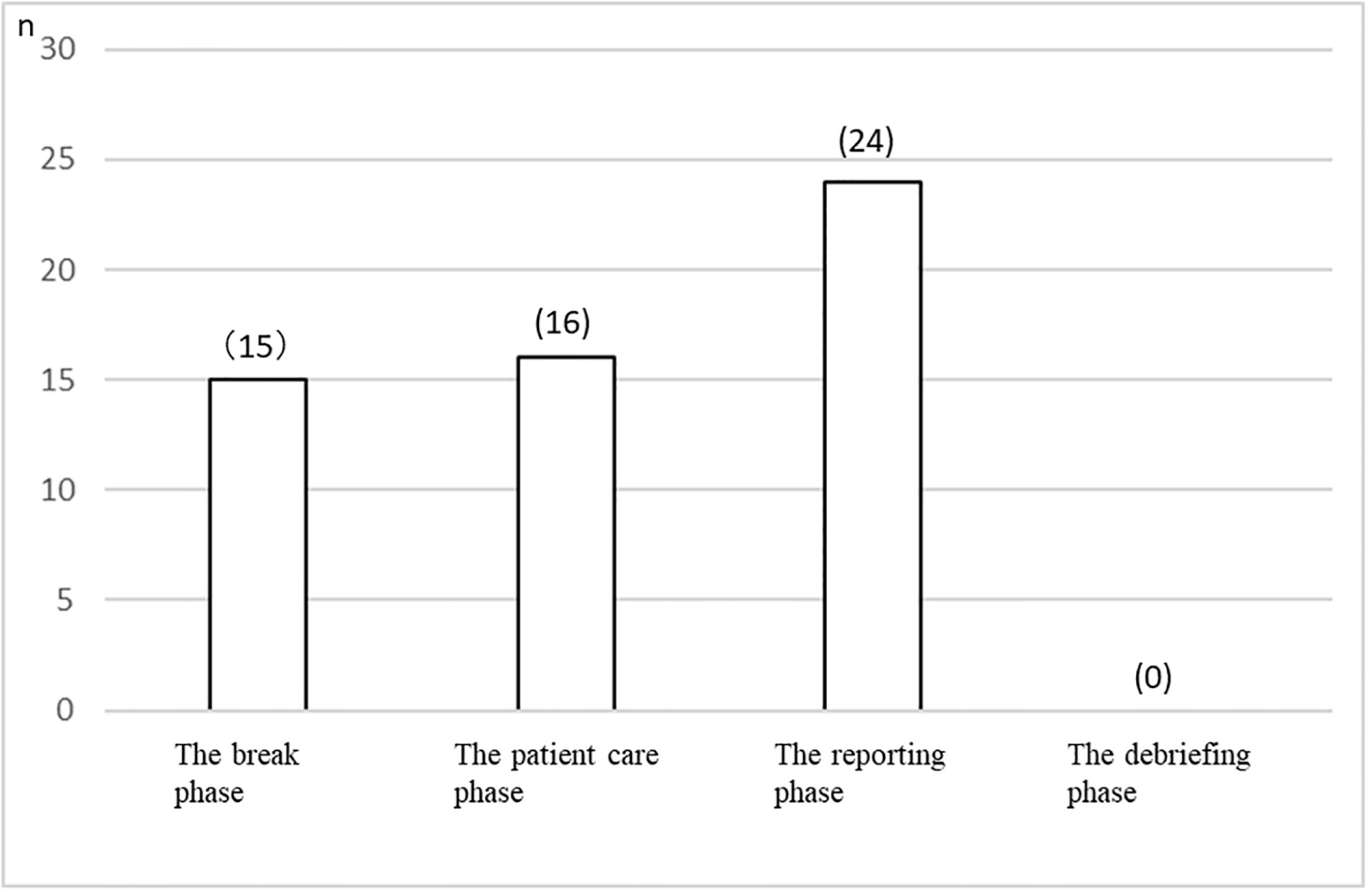
Students’ reports of the most stressful phase during the simulation (n = 55).

Twenty-four students who reported experiencing stress felt that they experienced the most stress during the reporting phase. Sixteen students answered that they most felt stressed during the patient care phase (Fig 4), while 18 students responded that they did not feel stressed. These last students said:

“*I thought it was a doll*.”

“*I calmed down because I was alone in the patient’s room*.”

One student said:

“*I couldn’t recognize whether I felt stressed*.”

## Discussion

The present study investigated changes in students’ HRV during simulation. We observed significantly lower HF and higher LF/HF in the reporting phase compared to the break phase. In addition, nearly half of the students who reported stress said that the most felt stressful phase was the reporting phase. Therefore, the reporting phase was characterized by high objective and subjective stress. Hamaideh et al. also examined the levels and types of stressors among 100 nursing students during their clinical training. They found that the presence of teachers and nursing staff was one of the greatest sources of stress during clinical training [18]. In this study, the presence of an educator was clear in the reporting phase, which might explain why students experienced such high stress during this phase. Labrague et al. conducted a systematic review on the levels of stress and main stressors for nursing students. They found that the stress levels of nursing students range from moderate to high, and that the main stressors included stress from caring for patients and negative interactions with staff and educators [19]. McEwen and Yerkes-Dodson’s laws have clarified that high stress can reduce performance [9, 20]. Thus, the high stress of the students in the reporting phase of this study might have influenced their reporting performance. The educators may need to look back on the behavior of students during the simulation, encourage students to think about it, and provide assistance in reporting. Ensuring that educators adopt supportive attitudes during this phase might help improve students’ performance.

In this study, we observed a significant reduction in HF during the patient care phase compared to the break phase. It became clear that the simulation stress was gradually increasing towards the reporting phase. On the other hand, many students reported feeling stress, and some felt that they were being watched, even though there was no one in the patient’s room. Even though it is assumed that the manner by which students express stress varies, how they experience stress varies as well. Li et al. showed that physical examination skills improved by simulation training more than traditional demonstration training [10]. In this study, it was not clear whether the stress that the student felt actually improved their skills or led them to have better performance during the simulation training. Still, educators should be aware that the stress of students will continue from the patient care phase to the reporting phase. We believe that if educators are cognizant of the progression of this stress, this will lead to a supportive attitude towards students at the reporting phase.

Although cardiac automaticity is intrinsic to various pacemaker tissues, heart rate and rhythm are largely under the control of the autonomic nervous system. The parasympathetic influence on heart rate is mediated through release of acetylcholine by the vagal nerve [16]. The understanding of the modulatory effects of neural mechanisms on the sinus node has been enhanced by spectral analysis of HRV. Vagal activity is the major contributor to the HF component [16]. However, spectral analysis of HF has other problems associated with breathing. HF oscillation corresponds to respiratory activity [21]. Breathing frequency during spontaneous breathing was distributed, with a median value of 0.25 Hz and a range between 0.18 Hz and 0.36 Hz [22]. Therefore, several studies have taken a way to paced breathing. Pagani et al. reported that paced breathing decreased LF, increased HF, and consequently decreased the LF/HF ratio [23]. There was no significant difference between HF power measured during spontaneous breathing and during paced breathing [24]. Therefore, although this study is on spontaneous respiration, there is a possibility that respiratory rate did not affect HRV. A significant HRV difference in this study can be attributed to stress as a major influencing factor. However, during mental stress, the breathing frequency increases [25]. Therefore, the impact of respiratory frequency in HRV is often unknown and further discussion is needed.

There are many studies showing that the practice of breathing at 6 breaths per minute can reduce stress, which is supported by HRV biofeedback. HRV biofeedback is a biobehavioral clinical intervention that has been gradually gaining empirical support for the treatment of psychological disorders and stress reduction [26,27]. McCraty and Shaffer (2015) reviewed that the use of real-time HRV feedback can improve self-regulatory capacity—namely, psychological resiliency and behavioral flexibility, which reflect an individual’s capacity to adapt to environmental demands [26]. Zwan et al.’s HRV biofeedback exercises had an overall beneficial effect, including reduced stress, anxiety, and depressive symptoms, as well as improved psychological wellbeing and sleep quality [27]. In this study, the changes in HRV due to stress during simulation were clarified. Specifically, students’ stress gradually increased across the simulation period. In revealing the phases of simulation that are most stressful to students, we might have provided an adequate target for HRV biofeedback, particularly for students who feel that they cannot report effectively because of their nervousness.

In this study, HF significantly decreased and LF/HF significantly increased in the reporting phase, and many students reported that the reporting phase was the most stressful. However, the debriefing phase was not significantly different from the break phase in terms of objective stress, and students said that they did not feel stressed. In other words, students are released from stress and their unfamiliar environment during this phase. This phase also gives them a chance to review/reflect upon their performance, which might help them understand how their own performance can be improved during the patient care and reporting phases due to high stress. Conventionally, the most important aspect of simulation is the debriefing phase (i.e., the review upon completion of the simulation). Lusk and Fater (2013) reported that the debriefing phase can improve students’ assessment capabilities by helping them develop a deeper understanding and reflect on the state of the patient, which they could not do during the reporting phase [28]. This study might have objectively confirmed the effects of debriefing as reported in previous studies using HRV. Similarly, Roh et al. (2016) surveyed the differences between instructor-led debriefing and peer-led debriefing, and found that the former led to better subsequent cardiopulmonary resuscitation performance among students, along with greater satisfaction with the simulation experience and higher ratings of debriefing quality [29]. Therefore, a high-quality debriefing is exceedingly important for ensuring students’ clinical performance and satisfaction with simulation. We believe that this statement may be better supported by triaging the results from students’ spoken or reported feedback on their emotions during each stage of the study. It might help educators to understand what emotions students experience during different stages of patient examination and care.

### Limitations and future directions

Given that students were all volunteers, we cannot rule out the possibility that students who participated were more willing to learn compared to students who did not participate. Thus, our findings may not be generalizable.

## Conclusion

In this study, we analyzed nursing students’ HRV during simulation and found that HF decreased from the break phase to the patient care phase, and then further to the reporting phase. On the other hand, LF/HF increased from the break phase to the reporting phase. In addition, 55 students felt stressed during the simulation, 24 of whom felt the most stressed during the reporting phase. Overall, this study provided both objective and subjective evidence of stress during postoperative simulation and further demonstrated the need to provide support at the reporting phase and the importance of debriefing.

## Supporting information

S1 Table DATA1 introduction ~ patient care. This is the value of the parameter at simulation phases from introduction to patient care.
(PDF)

S2 Table DATA2 reporting ~ debriefing. This is the value of the parameter at simulation phases from reporting to debriefing.
(PDF)
