# Supplementary material for: Heart rate variability can clarify students’ level of stress during nursing simulation
Source: PLoS One. 2018 Apr 5;13(4):e0195280. doi: 10.1371/journal.pone.0195280 (PMC5886456; doi:10.1371/journal.pone.0195280)
Supplement: S1 Table — This is the value of the parameter at simulation phases from introduction to patient care. (PDF) [file pone.0195280.s001.pdf]

ID	sex	age	introduction			patient care		
			HR	HF	LF/HF	HR	HF	LF/HF
1	1	20	93	97	4.2	102	125	3.9
2	1	20	64	415	2.8	78	866	3.2
3	0	21	90	107	5.9	104	104	6.5
4	0	21	70	581	2.3	80	233	4.6
5	0	21	81	22	8.4	79	48	14.2
6	0	21	72	472	1.4	74	279	3.1
7	0	22	69	289	5.3	68	152	4.3
8	0	21	98	136	12.8	110	54	7.9
9	0	20	67	760	2.1	67	1911	0.9
10	0	20	81	300	3.1	83	336	3.8
11	0	20	74	728	2.7	81	372	3.9
12	0	21	73	271	5.0	76	281	3.2
13	0	21	83	298	6.6	86	239	6.2
14	0	22	71	74	13.9	73	89	9
15	0	20	79	274	4.4	89	342	3.9
16	0	20	76	713	3.0	77	526	1.8
17	0	21	76	175	4.4	79	126	3
18	0	20	103	255	3.8	101	189	13.4
19	0	20	86	130	19.4	80	513	1
20	0	20	74	416	3.7	69	331	1.2
21	0	21	85	876	4.6	88	486	1.5
22	0	21	116	192	7.6	128	72	7.1
23	0	21	109	34	5.7	102	124	7
24	0	21	72	124	11.8	73	150	4.2
25	0	22	81	446	6.5	82	429	1.9
26	0	20	81	522	3.1	87	412	1.9
27	0	21	106	120	9.1	118	68	4.7
28	0	21	132	5	17	125	9	6.2
29	0	20	78	1087	2	85	330	4.4
30	0	21	85	129	10.8	83	138	7
31	0	20	98	134	2.7	101	74	4.2
32	1	22	100	350	3.4	102	161	5.1
33	0	22	72	401	1.4	75	389	1.6
34	0	21	80	542	2.6	87	453	3.4
35	1	21	95	1136	4.4	92	1450	6
36	1	22	88	226	3.7	98	145	2
37	1	27	78	879	1.2	86	380	2.8
38	0	20	77	580	3.5	89	434	4.8
39	0	20	87	405	3.7	91	232	3
40	0	20	83	151	1	81	501	2.4
41	0	21	94	283	1	107	140	2
42	0	20	79	324	2.4	70	1374	1
43	0	21	94	270	0.9	101	282	1.8
44	0	20	84	350	2.7	95	83	5.3
45	0	20	77	512	4.5	83	403	5.8
46	0	20	67	709	1.6	73	598	1.2
47	0	21	61	830	0.8	78	579	3.5
48	1	21	97	111	6.9	103	114	15.3
49	1	21	70	366	3.8	75	403	6.3
50	0	22	66	392	2.5	78	241	6.1
51	0	21	66	580	1.9	83	608	3
52	0	21	103	100	1.4	113	81	1.6
53	0	21	76	809	1.2	79	944	0.7
54	0	20	75	76	5	72	84	5
55	0	20	82	316	2.8	85	296	4
56	0	20	83	260	6.4	97	153	6.7
57	0	21	88	284	6.8	94	252	4.2
58	0	20	73	154	2.4	76	105	2.7
59	1	21	101	346	4.7	104	110	5.2
60	1	21	76	233	5.6	84	199	11.1
61	0	20	78	74	21.5	80	142	9.6
62	1	20	76	291	6.9	75	580	5
63	1	20	114	23	6.5	101	59	4.8
64	0	20	88	364	2.3	92	261	5.9
65	0	20	87	719	3.5	91	552	2.4
66	1	20	84	140	4	91	47	9.7
67	0	21	73	1130	0.5	76	706	2.8
68	0	21	85	440	10.1	90	272	3.2
69	0	21	75	512	2.2	73	489	3.3
70	0	21	86	633	2.5	85	487	2.9
71	1	21	78	765	4.9	80	679	4.9
72	0	21	75	266	4.5	83	104	12.2
73	0	21	82	161	1.6	94	81	3.1
74	0	21	90	398	4.2	97	303	4.7
