# Supplementary material for: Heart rate variability can clarify students’ level of stress during nursing simulation
Source: PLoS One. 2018 Apr 5;13(4):e0195280. doi: 10.1371/journal.pone.0195280 (PMC5886456; doi:10.1371/journal.pone.0195280)
Supplement: S2 Table — This is the value of the parameter at simulation phases from reporting to debriefing. (PDF) [file pone.0195280.s002.pdf]

ID	report			debriefing		
	HR	HF	LF/HF	HR	HF	LF/HF
1	103	48	7.7	96	89	4
2	70	445	4.4	69	426	5.3
3	103	29	8.4	86	119	4.7
4	84	391	2.6	73	179	13.7
5	84	34	23.9	87	20	28.1
6	84	262	3.3	80	253	13.9
7	111	45	20.1	73	249	0.5
8	96	101	8.4	101	91	10.3
9	83	590	3.4	71	1460	1.9
10	91	196	6.1	82	492	5.2
11	103	62	5.2	80	313	4.1
12	74	370	4.6	68	571	1.1
13	90	460	4.1	77	751	6.3
14	85	56	15	73	180	9
15	79	512	1.8	76	566	3.6
16	82	287	6	72	414	2.5
17	78	248	3.8	76	145	6.7
18	97	283	4.8	89	458	5
19	80	495	2	77	752	1.7
20	80	625	2.6	73	390	4.1
21	90	416	2.5	84	673	2
22	108	248	5.6	109	219	4.8
23	101	54	7	95	62	6
24	92	58	34	84	89	23.7
25	82	836	2.3	80	1094	2.3
26	89	164	3.6	84	432	2.7
27	120	20	3.5	108	93	3.7
28	136	8	13.5	126	41	6.7
29	87	143	7.1	75	651	2.8
30	93	88	7.1	91	573	2.6
31	86	132	3.9	90	150	3.5
32	104	124	10.2	92	276	8.5
33	72	622	3.4	72	510	3.4
34	93	273	5.7	75	622	4
35	110	307	3.3	83	405	1.3
36	105	250	3	84	383	2.1
37	86	380	2.8	84	211	2.3
38	90	368	6.1	74	1053	6.1
39	93	384	7.3	87	192	6.8
40	92	142	3.2	88	148	6.3
41	123	20	5.2	98	272	4.8
42	70	758	1.2	78	600	4.2
43	102	232	2.7	90	538	3.9
44	97	43	13.4	84	139	5.2
45	90	106	7.4	75	237	11.4
46	92	70	3.3	69	562	2.8
47	65	196	4.4	70	387	7.4
48	111	26	9.4	93	131	12.1
49	80	97	24.3	72	264	8.4
50	84	243	10.8	66	532	2.6
51	73	466	3.9	67	340	3.3
52	122	139	4.9	104	72	3.8
53	93	197	4.5	71	585	1.6
54	79	58	7.5	75	133	3.3
55	98	98	8	76	547	3.2
56	97	153	6.7	90	353	4
57	98	125	9.6	81	219	9.8
58	84	60	7.9	88	30	10.6
59	109	46	11.3	96	379	3.7
60	83	87	17.2	73	227	9
61	77	157	7.2	76	253	9.6
62	70	994	6	69	509	12.8
63	106	46	8.9	97	90	7.6
64	83	430	4	84	321	3.3
65	89	1279	2.3	87	986	3.3
66	81	36	21.2	83	117	2.7
67	68	868	1.3	75	483	2.7
68	95	288	7.7	91	220	9.9
69	87	197	4.2	71	1216	2.3
70	90	512	3.1	81	772	2.1
71	83	566	3.6	75	923	6.8
72	83	297	6.4	81	274	3.2
73	106	37	3.4	96	95	3.9
74	96	280	7.2	95	289	3.7
